# ART management in children perinatally infected with HIV from mothers who experience behavioural changes in Romania

**DOI:** 10.7448/IAS.17.4.19700

**Published:** 2014-11-02

**Authors:** Mariana Märdärescu, Alina Cibea, Cristina Petre, Ruxandra Neagu-Drãghicenoiu, Rodica Ungurianu, Sorin Petrea, Ana Maria Tudor, Delia Vlad, Carina Matei, Mãrdãrescu Alexandra

**Affiliations:** 1 Paediatric Immunodepression Department, Prof. Dr. Matei Bal, National Institute for Infectious Diseases, Bucharest, Romania; 2 Romanian HIV/AIDS Centre, “Prof. Dr. Matei Bal”, National Institute for Infectious Diseases, Bucharest, Romania

## Abstract

**Introduction:**

During the recent years the rate of HIV perinatally exposed children in Romania has increased as a consequence of the expanding number of HIV-infected women. These women belong to Romania's long-terms survivors, aged between 20 and 24 years and to the group of new HIV infection cases (20–24 years), acquired through unsafe sexual contact and use of new psychoactive substance (IV).

**Materials and Methods:**

We focused on 396 HIV perinatally exposed children born between 2008 and 2013, under surveillance in National Institute for Infectious Diseases “Prof. Dr. Matei Bals,” Bucharest. Of them, 43 acquired HIV through materno-foetal transmission. Our aim was to observe the characteristics in their evolution under antiretroviral treatment and to emphasize the causes of treatment failure. Children with perinatally acquired HIV infection were followed in a retrospective case series. We assessed maternal characteristics, HIV vertical transmission prophylaxis, timing of diagnosis, immunological and virologic status and features of the evolution under combined antiretroviral therapy (cART).

**Results:**

The rate of mother-to-child HIV transmission was 10.8% versus the national rate registered in 2013, namely <5%. 16% of mothers belonged to the Romanian 1990s cohort and 84% were recently infected with HIV, through unprotected sexual contact (70%) or use of new psychoactive substances (14%). 51% of mothers were diagnosed postnatally as a consequence of their reluctance to access specific health services and in 57% CD4 value was <350 cell/mm. 41% of the monitored children were diagnosed with HIV infection at birth. Their median entry CD4 value was 23% and 49% had a CD4 >25%; median entry viral load was 7 log. 16 patients (37%) had undetectable viral load after six months of treatment. In 87.5% of them the virologic suppression was achieved and maintained with one single regimen (2 NRTIs+1 NNRTI or 2 NRTIs+1 PI/r). 15 children (35%) did not achieve suppression of viral load. 19 children (44%) faced special issues related to adherence to antiretroviral treatment, due to mothers’ poor adherence to a basic set of cares destined for their children.

**Conclusions:**

Prevention programmes in Romania must be designed on the basis of the new economic context and emerging psychoactive substance use. Hence, women who use drugs should benefit from a wider access to medical and social services.
